# Au@Ag Core–Shell Nanorods Support Plasmonic Fano Resonances

**DOI:** 10.1038/s41598-020-62852-9

**Published:** 2020-04-03

**Authors:** Ovidio Peña-Rodríguez, Pablo Díaz-Núñez, Guillermo González-Rubio, Vanesa Manzaneda-González, Antonio Rivera, José Manuel Perlado, Elena Junquera, Andrés Guerrero-Martínez

**Affiliations:** 1 Instituto de Fusión Nuclear “Guillermo Velarde”, Universidad Politécnica de Madrid, Madrid, Spain; 2 Departamento de Ingeniería Energética, ETSII Industriales, Universidad Politécnica de Madrid, Madrid, Spain; 3 Departamento de Química Física, Universidad Complutense de Madrid, Madrid, Spain

**Keywords:** Chemistry, Materials science, Nanoscience and technology

## Abstract

In this work, we investigated experimentally and theoretically the plasmonic Fano resonances (FRs) exhibited by core–shell nanorods composed of a gold core and a silver shell (Au@Ag NRs). The colloidal synthesis of these Au@Ag NRs produces nanostructures with rich plasmonic features, of which two different FRs are particularly interesting. The FR with spectral location at higher energies (3.7 eV) originates from the interaction between a plasmonic mode of the nanoparticle and the interband transitions of Au. In contrast, the tunable FR at lower energies (2.92–2.75 eV) is ascribed to the interaction between the dominant transversal LSPR mode of the Ag shell and the transversal plasmon mode of the Au@Ag nanostructure. The unique symmetrical morphology and FRs of these Au@Ag NRs make them promising candidates for plasmonic sensors and metamaterials components.

## Introduction

Localized surface plasmon resonances (LSPRs) of metal nanoparticles, defined as collective oscillations of free electrons, have been extensively studied in recent years due to their versatility for a variety of applications, such as sensing, energy harvesting, and catalysis^1–5^. In particular, all-plasmonic Fano resonances (FRs) have attracted great interest during the past decade^6–8^. FRs are a type of resonant scattering phenomenon that gives rise to an asymmetric line-shape^9,10^. They have considerable potential for applications like waveguiding^11,12^, subwavelength optical imaging^13,14^, low-loss metamaterials preparation^12,15^, chemical and biological sensing^12,16,17^, and energy harvesting^18,19^, to name a few.

FRs are produced by the coupling of a discrete state with a continuum – e.g., between a narrow and a wide plasmon mode – and several plasmonic nanostructures have been proposed to display them^20^. Structural symmetry-breaking is the most common approach because it induces a non-uniform electromagnetic environment around the nanostructure, leading to the effective coupling between broad and narrow multipolar plasmon resonances. Examples of this approach are non-concentric multilayered nanoshells^21–23^, heterodimer nanostructures^6,24,25^, ring-disk nanocavities^26–28^, full nanocavities^29^, nanoparticle clusters^7,30–32^, and nanocrystals supported on substrates^16,33^. The main disadvantage of this strategy is that the complex and/or asymmetric nanostructures are fabricated using intricate and expensive techniques^27^, and/or only work under specific conditions^34^, which largely reduce their applicability.

In contrast, the generation of plasmonic FRs in highly symmetric metal nanoparticles is much more challenging; indeed, only a few examples of them exist, such as bimetallic nanoparticles^25,35,36^, metallic nanoshells^8,21^, and in some metal@dielectric^37–39^ an all-dielectric^40^ core–shell nanostructures. However, these systems are easier to fabricate and are thus more attractive from the application point of view. In this work, we report the observation of two different FRs on core–shell nanorods (NRs) composed of a Au core and a Ag shell (Au@Ag NRs). In this system, the spectrally localized LSPR modes of the Ag shell (the discrete levels) couple to the Au interband transitions (the continuum), showing remarkable tunability of the FRs. Although these Au@Ag NRs had been previously synthesized by a colloidal seed-mediated method^41,42^, their plasmonic modes had not been identified so far as FRs.

## Results and Discussion

**Figure 1:**
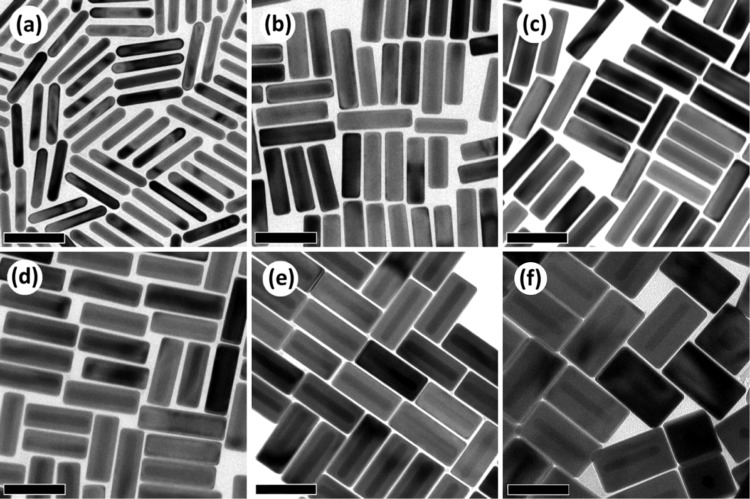
Low-magnification TEM micrographs of Au NRs (**a**) and Au@Ag NRs (**b–f**) with increasing thicknesses of the Ag shell. The dimensions of the nanoparticles are shown in Table 1. The scale bars in the images represent 100 nm.

In order to investigate the role of Ag overgrowth over Au NRs on the emergence of FRs, Au@Ag NRs with different amounts of Ag were synthesized (see Methods section), as shown in Fig. 1. The cuboid morphology displayed by the Au@Ag NRs had already been previously observed due to stabilization of the {100} facets of Ag ffc nanocrystals^41^. The dimensions and composition of the different nanostructures are collected in Table 1.

**Table 1:** Longitudinal LSPRs and dimensions of the Au@Ag NRs and the Au NRs used to seed their growth.

Nanoparticle	Longitudinal LSPR (nm)	Length (nm)	Width (nm)	Aspect Ratio	Au%	Ag%
AuNR	1000	102 ± 5	18 ± 3	5.8	100	0
Au_24_@_76_Ag NR	800	112 ± 7	30 ± 4	3.8	24	76
Au_19_@Ag_81_ NR	770	111 ± 7	34 ± 4	3.3	19	81
Au_14_@Ag_86_ NR	750	118 ± 9	38 ± 5	3.1	14	86
Au_8_@Ag_92_ NR	715	118 ± 9	50 ± 6	2.4	8	92
Au_4_@Ag_96_ NR	700	125 ± 10	74 ± 7	1.7	4	96

The volume percentages given in the last two columns represent, respectively, the Au and Ag content in the different Au@Ag NRs.

**Figure 2:**
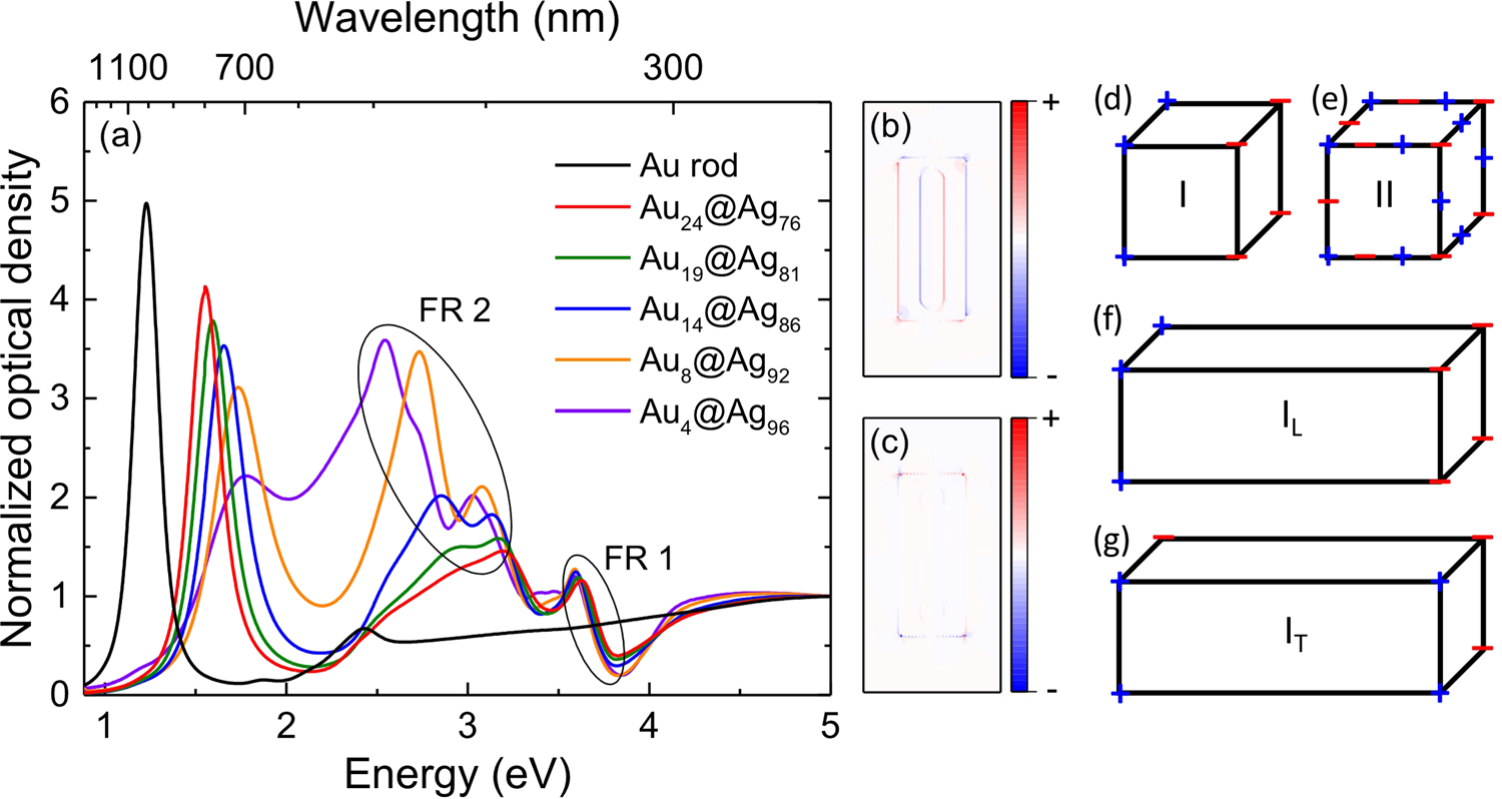
(**a**) Experimental spectra obtained for the Au@Ag NRs depicted in Fig. 1, where FR1 and FR2 are marked. The sub-indexes in the legend represent the percentage in volume for each material in the nanostructure (Table 1). (**b**,**c**) Charge distribution in a cut along the xy plane, calculated for FR1 and FR2, respectively. (d,e) Schematic surface charge distributions of the two main LSPR modes of the cubic morphology. Schematic surface charge distributions for the (**f**) longitudinal and (**g**) transversal modes of plasmon mode I when the cube is elongated in one direction.

The optical response of these nanostructures is depicted in Fig. 2. Au NRs exhibit the typical LSPRs, with one peak located at 1000 nm and the other one at 510 nm, corresponding to the longitudinal and transversal plasmon modes, respectively. On the other hand, Au@Ag NRs present richer plasmonic features. For instance, the presence of the longitudinal plasmon mode can still be observed, but it blue-shifts with the increasing Ag content (i.e., with the decreasing aspect ratio, see Table 1). More interestingly, two clear FRs arise, with approximate spectral locations at 3.7 eV (335 nm) and 2.92 (425 nm)–2.75 eV (450 nm); hereafter, we will refer to them as FR1 and FR2, respectively. The typical charge distribution is very different for both FRs, as can be seen in Fig. 2b,c.

To better understand the origin of the FRs, we have to discuss first the LSPR modes of the cubic morphology. A cube sustains an infinite number of plasmon modes^16^, but six of them account for 96% of the total oscillator strength^43^. Indeed, the FRs observed in the Au@Ag NRs can be ascribed to the two LSPR modes with the lowest energy, which represent almost 70% of the total oscillator strength^43^, as the other modes fall in the region of interband transitions of Ag. The charge distribution for these two main modes is represented schematically in Fig. 2d,e. In both modes, charges are concentrated on the corners of the cube^43,44^, with charges of the same sign located on opposite sides. However, mode II presents also charges of opposite sign located on the edges of the cube^16^. Both modes have a large electric dipole moment and couple strongly to light, with oscillator strengths of 0.44 and 0.24 for plasmon modes I and II, respectively^43^. However, the dominant mode (I) exhibits remarkable tunability compared to the other mode^16^. This is particularly true when the cube is elongated in one direction^45^; the main plasmon splits into a longitudinal and a transversal mode (Fig. 2f,g), with the former red-shifting as a function of the aspect ratio while the displacement for the latter is practically null.

**Figure 3:**
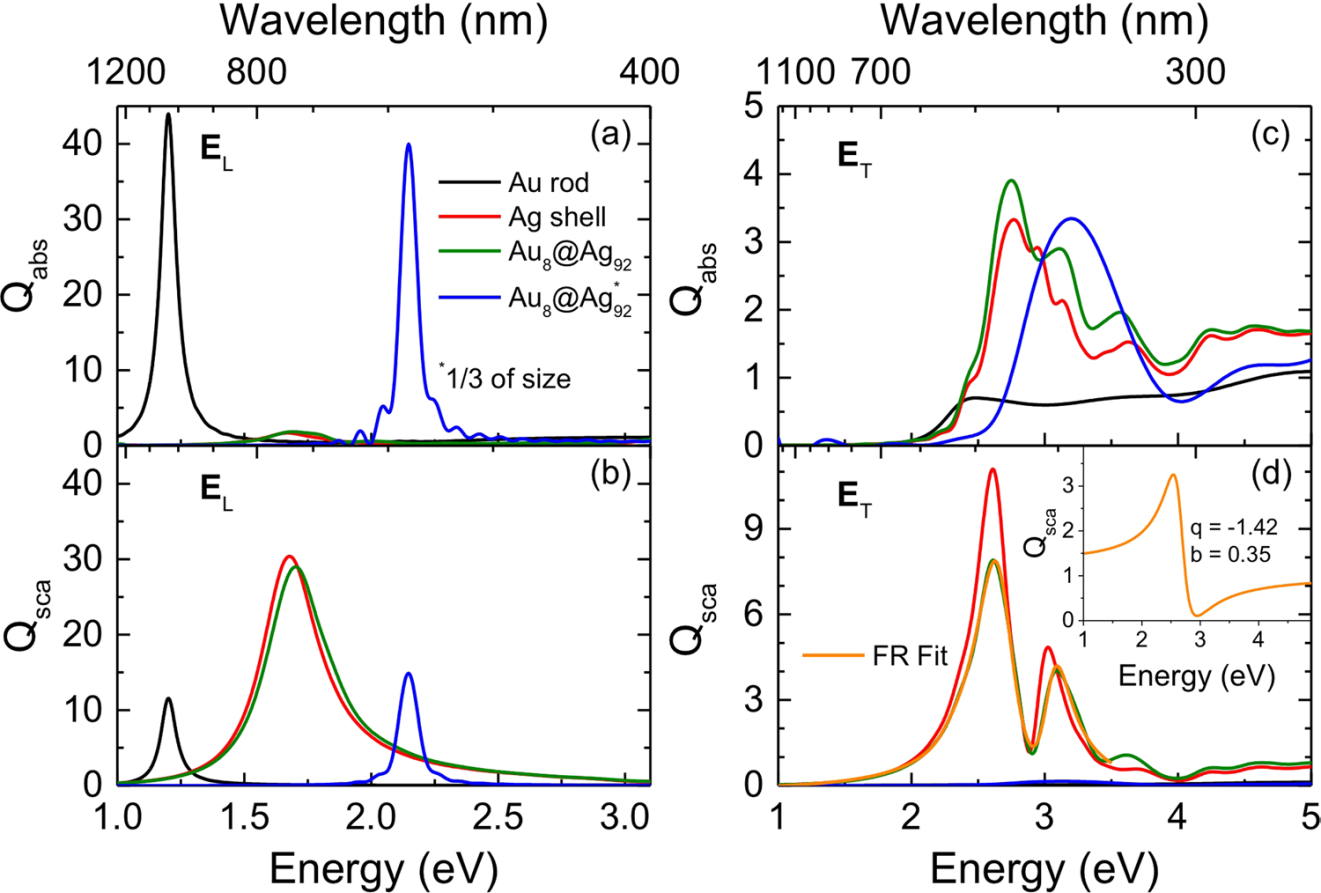
(**a**,**c**) Absorption and (**b**,**d**) scattering efficiencies obtained from FDTD simulations, with the electric field polarized along either the (**a**,**b**) longitudinal or the (**c**,**d**) transversal direction. The spectra were calculated for the Au NRs, the silver shell, the whole Au@Ag NR, and a small Au@Ag NR (with dimensions reduced to 1/3 with respect to the original particle). The fit of the FR profile (orange line) is shown in (**d**), where the inset shows the isolated profile of the FR.

Figure 3 shows the FDTD simulations performed to help us understand the origin of the FRs. No FR is observed when the light is polarized along the longitudinal axis of the nanoparticles (Fig. 3a,b) whereas both FRs appear with transversal polarization (Fig. 3b). FR1 only appears in the Au@Ag NRs (Fig. 3c,d), which suggests that it is caused by the interaction between a plasmonic mode of the nanoparticle and the interband transitions of Au. In fact, this FR is very similar to that observed previously for a spherical core–shell plasmonic nanostructure, ascribed to coupling of the Ag shell anti-bonding mode, which corresponds to the negative parity of the dipoles; i.e., the antisymmetric field (see Fig. 2b)^25^.

On the other hand, FR2 is present for both the Au@Ag NRs and the Ag shell. Hence, it can be ascribed to the interaction between the dominant transversal LSPR mode of the Ag shell and the transversal plasmon mode of the whole nanostructure. This latter transversal mode can be clearly seen in Fig. 3c,d for a nanostructure with identical aspect ratio but with the dimensions reduced to 1/3 those of the original particle. The scattering is negligible for this small particle and, hence, the transversal plasmon mode is observed without the FR.

In order to obtain additional insights into the characteristics of FR2, we fitted it with the expressions developed by Gallinet and Martin for FRs on a continuum-like wide LSPR mode having a Lorenzian shape^20,46^:

1 $${Q}_{{\rm{sca}}}(E)=\frac{{a}^{2}}{\left(\frac{{E}^{2}-{E}_{{\rm{s}}}^{2}}{2{E}_{{\rm{s}}}{\Gamma }_{{\rm{s}}}}\right)+1}\times \frac{{\left(\frac{{E}^{2}-{E}_{{\rm{a}}}^{2}}{2{E}_{{\rm{a}}}{\Gamma }_{{\rm{a}}}}+q\right)}^{2}+b}{{\left(\frac{{E}^{2}-{E}_{{\rm{a}}}^{2}}{2{E}_{{\rm{a}}}{\Gamma }_{{\rm{a}}}}\right)}^{2}+1}$$

The two terms of Eq. (1) represent, respectively, the symmetric pseudo-Lorentzian line shape (subscript ‘s’) and the Fano-like asymmetric line shape (subscript ‘a’). In this equation, *a* is the maximum amplitude of the Lorentzian resonance, *E*_s_ is the resonance energy position, and *Γ*_s_ is its approximate spectral width. Likewise, for the asymmetric FR, *E*_a_ is the position of the resonance center, *Γ*_a_ gives an approximation of its spectral width, *q* is the asymmetry parameter, and *b* is the modulation damping parameter originating from intrinsic losses. To better fit our spectra, we added a sigmoidal term [*B*_1_ + *A*_1_/(1 + exp(−*A*_2_*(*E* − *E*_0_)))] to account for the interband absorption of Au^8^. Here *B*_1_, *A*_1_, *A*_2_, and *E*_0_ are the offset, amplitude, slope, and position of the sigmoid, respectively. As can be seen in Fig. 3d, the fit of the Fano profile is excellent. The isolated profile of the FR (i.e., the asymmetric term) is shown in the inset of Fig. 3d. This clearly confirms that the features of the spectrum of Au@Ag NRs are dominated by two FRs, one of them produced by the interaction of a plasmonic mode of the Ag shell with the interband transitions of Au and the other by the interaction between two plasmonic modes of the whole Au@Ag NRs.

**Figure 4:**
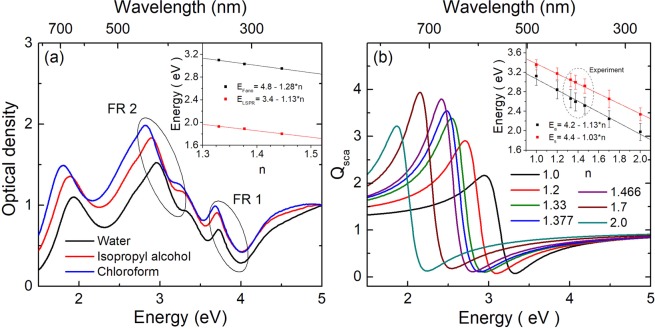
(**a**) Experimental optical spectra of Au_8_@Ag_92_ NRs immersed in different media: water (black line, *n* = 1.33), isopropyl alcohol (red line, *n* = 1.377) and chloroform (blue line, *n* = 1.466). (**b**) Fano resonance, extracted from a fit of the theoretical optical spectra, calculated for the Au_8_@Ag_92_ NR with transversal polarization and immersed in media with different refractive indexes. In both figures, the insets show the position of FR2 and the LSPR for the given conditions.

Finally, we have studied the behavior of the FRs as a function of the refractive index of the medium, to evaluate their potential use as sensors (Fig. 4). First, as depicted in Fig. 4a, we dispersed Au_8_@Ag_92_ NRs in three different media (see Methods section): water (*n* = 1.33), isopropyl alcohol (*n* = 1.377) and chloroform (*n* = 1.466). As can be seen in the inset of this figure, FR2 and the longitudinal plasmon mode exhibit a very similar dependence on *n*, but is slightly more sensitive for the former. FR1, on the other hand, shows no appreciable variations on position or shape for the different media. Moreover, we performed FDTD simulations of the optical response of the same Au@Ag NR for light polarized transversal to the nanostructure and different refractive indices (between 1 and 2, including the experimental values). From a fit to this data with the model described by Eq. (1), we extracted the position and shape of FR2 (Fig. 4b) and the transversal LSPR mode (not shown). As can be seen in the inset of Fig. 4b, their shift is very similar but, again, it is slightly larger for FR2. Hence, we can conclude that for these nanostructures FR2 is slightly better than the LSPR for sensing applications whereas FR1 is almost independent of the environment.

## Conclusions

In summary, we have shown that a nanostructure composed of a Au NR covered with a silver shell is a very simple symmetrical nanoparticle that exhibits two different FRs. In addition to geometric simplicity, these nanoparticles are easy to synthesize using a standard colloidal method. Therefore, the configuration we are proposing is a very advantageous alternative to previous systems exhibiting FRs, surpassing them in simplicity, tunability, and intensity of the resonances. We have also shown that the tunable FR located at lower energies is strongly dependent on the refractive index of the surrounding medium. This may open the door to a number of applications, such as in sensing, where FRs have often been proposed as a good alternative to LSPRs but which have been seldom realized experimentally due to the complexity of the systems able to generate intense FRs.

## Methods

### Colloidal synthesis

#### Chemicals

All the products were obtained from Sigma Aldrich, namely, cetyltrimethylammonium bromide (CTAB, ≥99%), cetyltrimethylammonium chloride (CTAC, 25% w/w aqueous solution), *n-*decanol (98%), gold(III) chloride trihydrate (HAuCl_4_·3H_2_O, ≥99.9%), nitric acid silver(I) salt (AgNO_3_, ≥99.0%), L-ascorbic acid (≥99%), and sodium tetrahydridoborate (NaBH_4_, 99%). Deionized water was used in the syntheses of nanoparticles (resistivity 18.2 MΩ cm at 298 K). The organic solvents with spectrophotometric grade and poly(ethylene glycol) methyl ether thiol (*M*w = 6000) were purchased from Sigma-Aldrich.

#### Synthesis of Au NRs

The Au NRs were prepared using a seeded growth method with some modifications^47^:

##### Synthesis of 1–2 nm au seeds

200 µL of a 0.05 M HAuCl_4_ solution and 100 µL of a 0.1 M ascorbic acid solution were added under stirring (ca. 500 rpm) to 20 mL of a 50 mM CTAB and 13.5 mM *n-*decanol solution in a 50 mL glass beaker. The temperature was maintained between 25 and 27 °C. After 1–2 min, 800 µL of a freshly prepared 0.02 M NaBH_4_ solution was injected into the previous colorless solution under vigorous stirring (1000 rpm using a PTFE plain magnetic stirring bar: 30 × 6 mm), affording a brownish-yellow solution. The seed was aged for 1 h at 25–27 °C prior to use.

##### Synthesis of small anisotropic seeds (21 nm in length and 7.5 nm in width)

In a typical synthesis, 300 mL of a 50 mM CTAB and 11 mM *n-*decanol solution was placed in a 500 mL Erlenmeyer and 3000 µL of 0.05 M HAuCl_4_, 2400 µL of 0.01 M AgNO_3_, 21 mL of 1 M HCl, and 3900 µL of 0.1 M ascorbic acid were sequentially added. The temperature of the solution was maintained at 25 °C. Then, 18 mL of the 1–2 nm seed solution was added under stirring. The mixture was left undisturbed at 25 °C for at least 4 h until the solution changed from colorless to dark brownish gray. The obtained small anisotropic seeds (longitudinal LSPR located at 725–730 nm) were centrifuged at 14000–15000 rpm for 60 min. The precipitate was redispersed with 100 mL of 10 mM CTAB solution and centrifuged under the same conditions. The final Au concentration was fixed to 4.65 mM (Abs_400nm_: 1, optical path: 0.1 cm).

##### Au NRs with LSPR at 1000 nm

In a typical synthesis, 2500 µL of 0.01 M AgNO_3_, 1000 µL of 0.05 M HAuCl_4_, 9000 µL of 1 M HCl, and 800 µL of a 0.1 M ascorbic acid solution were added under stirring to 100 mL of a 50 mM CTAB and 11 mM *n-*decanol solution at 28 °C. Then, 0.5 mL of the small anisotropic seed suspension was added under stirring. The mixture was left undisturbed for 6 h. The Au NRs were forced to settle as sediment (by centrifugation at 7000 rpm, 30 min) to remove the excess of surfactant and redispersed in 10 mL of a 25 mM CTAC solution (Au NR stock solution). This procedure was repeated twice to remove CTAB traces. Finally, they were redispersed in 2.5 mL of a 25 mM CTAC solution. The resulting Au NRs presented an average length of 102 ± 5 nm and diameter of 18 ± 2 nm.

#### Synthesis of Au@Ag NRs

The nanoparticles were prepared following a seeded growth method with some modifications^41^. Briefly, 500 µL of 0.01 M AgNO_3_ and 50 µL of a 0.01 M ascorbic acid solution were added to 10 mL of a 25 mM CTAC solution. The concentration of Au NRs in the growth mixture was varied to obtain different Ag shell thicknesses (Table 2). Then, the mixture was heated up to 65 °C and left undisturbed for 12 h. Finally, the nanoparticles were centrifuged for 30 min at 5000 rpm and redispersed in 10 mL of a 25 mM CTAC solution.

**Table 2:** Volume of Au NR stock solution added to seed the growth of Au@Ag NRs and the resulting Au concentration in the growth mixture.

Nanoparticle	Longitudinal LSPR (nm)	Volume of AuNRs (μL)	Concentration of Au (mM)
Au_24_@_76_Ag NR	800	60	0.12
Au_19_@Ag_81_ NR	770	47.5	0.095
Au_14_@Ag_86_ NR	750	34.8	0.07
Au_8_@Ag_92_ NR	715	20	0.04
Au_4_@Ag_96_ NR	700	10	0.02

#### Polyethylene glycol functionalization of Au@Ag NRs

An aqueous solution of poly(ethylene glycol) methyl ether thiol (6 kDa) previously sonicated for 5 min was added to Au@Ag NRs (1 nM, 5 mL) stabilized with CTAC (1 mM) under stirring for 3 h. Then, the excess of free ligand was removed from the solution by four centrifugation cycles (6500 rpm, 90 min). In each cycle, the supernatant was removed and the precipitate was redispersed in the same volume of solvent. The order of solvent transfer was water, isopropyl alcohol and chloroform.

#### Transmission electron microscopy (TEM)

Low magnification TEM images were obtained on a JEOL JEM-1400PLUS transmission electron microscope operating at an acceleration voltage of 120 kV. Carbon-coated 400 square mesh copper grids were used. For TEM grid preparation, 1.5 mL of the mixture was centrifuged (in 1.5 mL Eppendorf tubes) and redispersed in 1.5 mL of a 1 mM CTAC solution. Then, the nanoparticles were centrifuged again (same parameters) and redispersed in 20 μL of a 1 mM CTAC solution. Finally, 3 μL of water and 1 μL of the nanoparticle suspension were deposited on a carbon-coated 400 square mesh copper grid (placed on Parafilm) and allowed to dry slowly.

#### UV–vis–NIR Spectroscopy

Extinction spectra were recorded on a UVICONXL spectrophotometer (Bio-Tex Instruments). All experiments were carried out at 298 K using quartz cuvettes with an optical path length of either 2 mm or 1 cm.

### Optical Simulations

**Figure 5:**
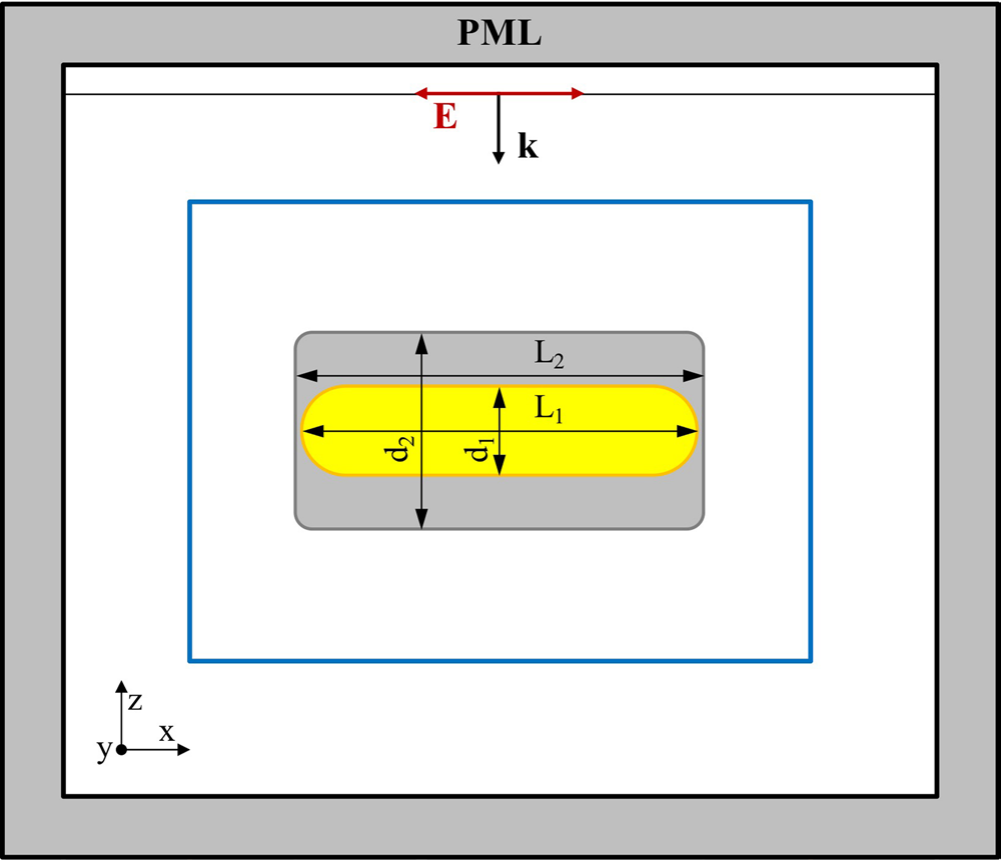
2D schematic representation of the 3D model used to simulate the optical response of the core–shell nanoparticles. An *x*-polarized plane wave impinges over the nanostructure and the flux is measured on all the faces of a parallelepiped containing the whole nanostructure (the blue rectangle) to determine the scattering, absorption, and extinction efficiencies (*Q*_sca_, *Q*_abs_, and *Q*_ext_, respectively). The simulation is repeated three times, with the plasmonic nanoparticle aligned along the three Cartesian axes. The simulation area is surrounded by a perfectly matched layer (PML) to mimic an infinite space.

Optical response and near-field enhancements were calculated using the method of finite differences in the time domain (FDTD), as implemented in the free software package MEEP^48^. In this method, Maxwell equations are solved by a second-order approximation. Space is divided into a discrete grid, the Yee grid^49^, and the fields are evolved in time using discrete time steps. A schematic representation of the geometry used for the calculation is shown in Fig. 5. Simulations were performed for the nanostructures oriented along the three Cartesian axes. In all calculations, we employed a spatial resolution of 0.5 nm. For the refractive index, we applied the bulk values of Ag and Au fixed by Johnson and Christy^50^, using a Drude term and five Lorentzians^51^. For the calculations in Fig. 3, the refractive index of the surrounding medium was fixed at 1.33 (water) whereas different values between 1 and 2 where used to study the sensing capabilities of the FRs. In Fig. 5, *d*_1_ and *d*_2_ are, respectively, the rod diameter and the box side. Likewise, *L*_1_ and *L*_2_ represent the length of the rod and the box, respectively. The edges of the box were rounded with a radius of 2 nm, to make it more similar to the experimental structures (see Fig. 1).

## Data Availability

The data generated and analysed during the current study will be made available from the corresponding author on reasonable request.
